# Supplementary material for: Dynamic Change of Volatile Fatty Acid Derivatives (VFADs) and Their Related Genes Analysis during Innovative Black Tea Processing
Source: Foods. 2024 Sep 28;13(19):3108. doi: 10.3390/foods13193108 (PMC11475071; doi:10.3390/foods13193108)
Supplement: Supplementary file 1 [file foods-13-03108-s001.zip › Figure S1.pdf]

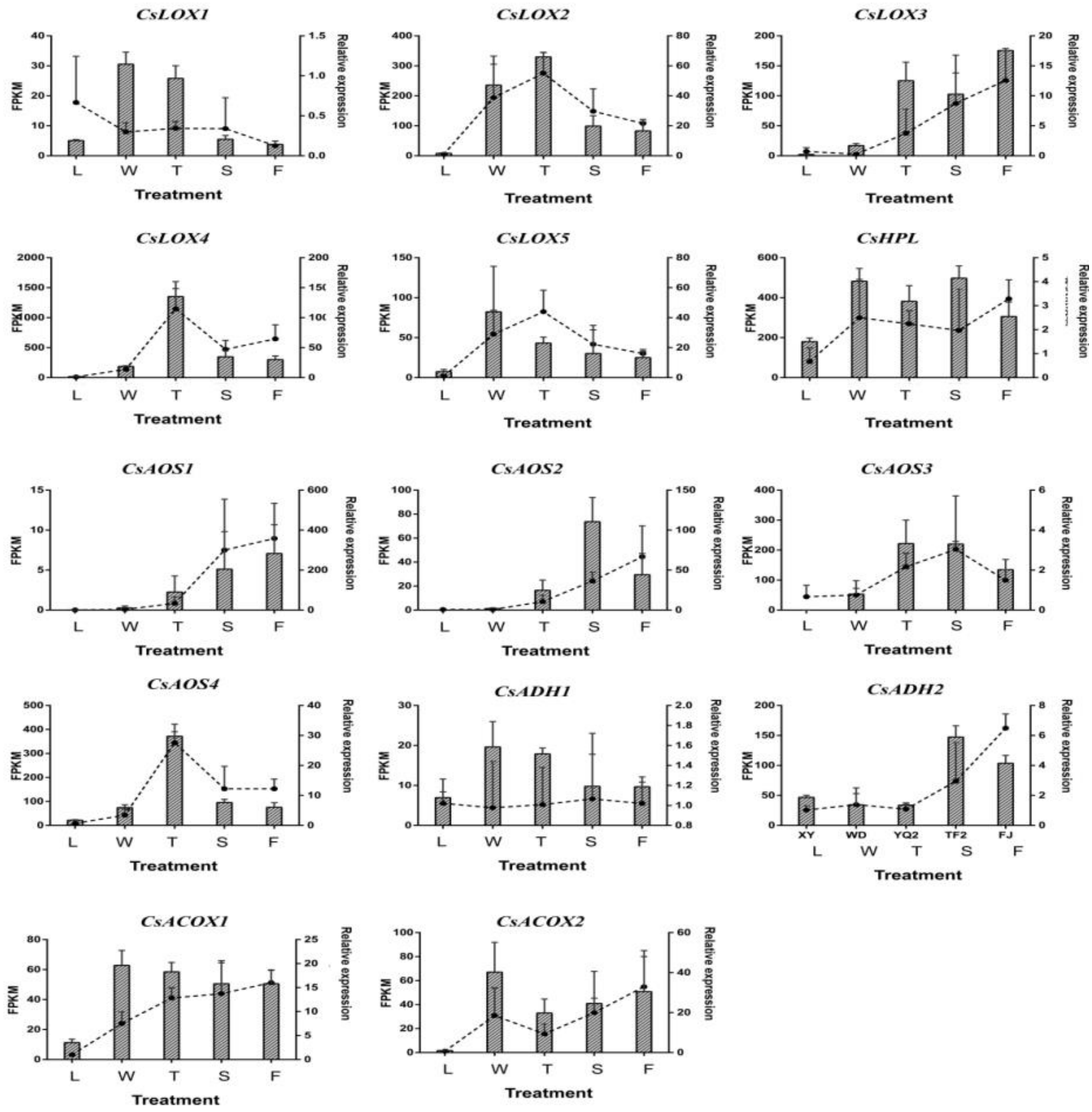

Figure S1. FPKM value and RT-qPCR verification of fatty acid metabolic pathway genes. FPKM: Fragments Per Kilobase of exon model per Million mapped fragments. L: fresh leaves, W: after solar-withering, T: after second time turn-over, S: after second time spreading, F: after fermentation. Column chart represents FPKM value. The dashed line represents the validation result of qRT-PCR. Error bars indicate the standard error (SE) of the mean.
