# Supplementary material for: Dynamic Change of Volatile Fatty Acid Derivatives (VFADs) and Their Related Genes Analysis during Innovative Black Tea Processing
Source: Foods. 2024 Sep 28;13(19):3108. doi: 10.3390/foods13193108 (PMC11475071; doi:10.3390/foods13193108)
Supplement: Supplementary file 1 [file foods-13-03108-s001.zip › Table S1.pdf]

Table S1. qRT-PCR to verify the primer information of genes

Gene Name	transcript ID or accession number	Forward primer sequence (5′–3′)	Reverse primer sequence (5′–3′)	Annealing temperature (°C)
CsLOX1	TGY077916	ATTTGGGTGCCTCGGTT	TGTCTGTGGTGGTGGTTTC	62.1
CsLOX2	TGY094953	GACGGGATTAGAGAAGGAAAC	CAAGTGACATCAACAGGGC	60.9
CsLOX3	TGY024305	CCCAATGCCACAAGTGAT	GGCTTCAAAGTCCCGTCTT	61.0
CsLOX4	TGY017079	GGAGGACTTGAAGGAGACACT	CCACTGTGAACTCTGCTATGTAG	63.4
CsLOX5	TGY094955	TGGAAGAGACGGGATTAGAG	CAAGTGACATCAACAGGGC	61.2
CsADH1	TGY037356	TCCTGTTCCTGCTCCAA	CAACTACTTCTCCTGCTACATCAG	60.7
CsADH2	TGY097376	CTCGCAAACTTCCTTTCAC	GAACTGTCATTGTCTCGTTAGC	59.8
CsAOS1	TGY084375	AATCTATGCTTCCGTATCCG	CGAGTCTGTCTTTGATGGG	60.1
CsAOS2	TGY084373	ATCCGAAAGACCATCTGC	GAAATGAAGGGACCAGGTG	59.9
CsAOS3	TGY021554	TCTTCTTCATCTCCACCACA	GGGAATGTTTAGGTTCGGA	60.0
CsAOS4	TGY031841	CGATTACTTCTACAACCAGGG	AGTTTGGCGTGATTGGG	60.6
CsACOX1	TGY062748	TCTCTCCCGATTACAACCA	CATAGAAAGAAGTGGACGCC	60.7
CsACOX2	TGY007218	ATCCAACGGCTGTCCTT	CTCTCTTGTATGTCCCTGTGC	61.5

CsHPL	TGY044402	ACCCTCACCATCCCTAACA	CCTTGGAACCAGAAGTAGTCG	63.1
CsGADPH	KA295375.1	TTGGCATCGTTGAGGGTCT	CAGTGGGAACACGGAAAGC	64.1
