# Supplementary material for: Dynamic Change of Volatile Fatty Acid Derivatives (VFADs) and Their Related Genes Analysis during Innovative Black Tea Processing
Source: Foods. 2024 Sep 28;13(19):3108. doi: 10.3390/foods13193108 (PMC11475071; doi:10.3390/foods13193108)
Supplement: Supplementary file 1 [file foods-13-03108-s001.zip › Table S2.pdf]

Table S2 The information of 45 identified volatile fatty acid derived compounds

No	Compounds	CAS	NIST_RI	Odor	Threshold	Class
1	(*E*)-6-Nonenal	2277-20-5	1124	-	2.20E-05	Aldehyde
2	(*Z*)-7-Decen-1-al	21661-97-2	1212	citrus, aldehydic, cucumber	2.20E-03	Aldehyde
3	(*E*)-2-Decenal	3913-81-3	1263	waxy, fatty, earthy, green	5.00E-03	Aldehyde
4	(*Z*)-2-Decenal	2497-25-8	1252	tallow	5.00E-02	Aldehyde
5	(*E,E*)-2,4-Nonadienal	5910-87-2	1216	fatty, melon, waxy, green	1.60E-04	Aldehyde
6	(*2E,4Z*)-2,4-Decadienal	25152-83-4	1295	fried, fatty, green	7.00E-05	Aldehyde
7	(*E*)-4-Decenal	65405-70-1	1198	fresh, aldehydic, citrus	2.50E-02	Aldehyde
8	(*Z*)-4-Heptenal	6728-31-0	900	oily, fatty, green, dairy	2.50E-05	Aldehyde
9	Hexanal	66-25-1	801	aldehyde, grassy, green	5.00E-03	Aldehyde
10	Tridecanal	10486-19-8	1513	fresh, clean, aldehydic	7.00E-02	Aldehyde
11	(*E,E*)-2,4-Undecadienal	30361-29-6	1430	oily, caramel, spicy, citrus,	1.00E-03	Aldehyde
12	(*Z*)-2-Penten-1-ol	1576-95-0	768	green, phenol, nasturtium	7.20E-01	Alcohol
13	(*E*)-3-Nonen-1-ol	10339-61-4	1143	green, waxy, melon	-	Alcohol
14	(*E*)-2-Decen-1-ol	18409-18-2	1257	waxy, fresh air, citrus, rose		Alcohol
15	1-Decanol	112-30-1	1272	fatty, waxy, floral, orange,	2.30E-02	Alcohol
16	2,4-Decadien-1-ol	14507-02-9	1274	fatty, waxy, citrus, melon		Alcohol
17	n-Tridecan-1-ol	112-70-9	1577	musty	-	Alcohol
18	1-Octanol	111-87-5	1072	intense citrus, rose	2.20E-02	Alcohol
19	1-Nonen-4-ol	35192-73-5	1103	-	-	Alcohol
20	(*E*)-2-Nonen-1-ol	31502-14-4	1169	waxy, green, violet, melon	2.09E-01	Alcohol
21	Hexanoic acid, 1-methylethyl ester	2311-46-8	1034	fruity, pineapple	-	Ester
22	(*Z*)-4-Decenoic acid, methyl ester	7367-83-1	1323	fruity, pear, mango, fishy	3.00E-03	Ester
23	Formic acid, octyl ester	112-32-3	1114	fruity, rose, orange, waxy	-	Ester
24	Acetic acid, octyl ester	112-14-1	1210	green, earthy, mushroom	1.40E-01	Ester
25	n-Amyl isovalerate	25415-62-7	1110	apple, fresh fruit	1.20E+01	Ester
26	Butanoic acid,2-methyl-, 2-methylpropyl ester	2445-67-2	1004	sweet, fruity	4.30E-02	Ester
27	Nonanoic acid, ethyl ester	123-29-5	1295	fruity, rose, waxy, rummy,	1.00E-02	Ester
28	Heptanoic acid, ethyl ester	106-30-9	1098	fruity, pineapple, cognac	2.00E-03	Ester
29	Hexanoic acid, 3-hexenyl ester	31501-11-8	1380	fruity, green, waxy, pear	7.81E-01	Ester
30	Octanoic acid, methyl ester	111-11-5	1126	fruity, citrus	2.00E-01	Ester
31	Isobutyl isovalerate	589-59-3	1005	sweet, fruity, apple, raspberry	3.40E-02	Ester
32	Hexanoic acid, butyl ester	626-82-4	1192	fruity, pineapple, berry, apple	7.00E-01	Ester
33	(*E*)-butanoic acid, 3-hexenyl ester	53398-84-8	1185	-	-	Ester
34	Butanoic acid, 5-hexenyl ester	108058-75-9	1183	-	-	Ester
35	Hexanoic acid, methyl ester	106-70-7	925	ethereal, fruity, pineapple, apricot	7.00E-02	Ester
36	Hexanoic acid, 5-hexenyl ester	108058-81-7	1371	-	-	Ester
37	2-Butenoic acid hexyl ester	19089-92-0	1191	sweet, caramel, green, walnut, oily	-	Ester

38	Ethyl 5-methylhexanoate	10236-10-9	1068	-	-	Ester
39	Butanoic acid, octyl ester	110-39-4	1393	fresh, waxy, fruity, green	2.50E-01	Ester
40	Butanoic acid, 3-methylbutyl ester	106-27-4	1056	fruity, green, apricot, pear, banana	7.00E-02	Ester
41	Butyl caprylate	589-75-3	1389	buttery, ether, herbal, dank	-	Ester
42	Butanoic acid, 2-methyl-, hexyl ester	10032-15-2	1236	green, waxy, fruity, apple, spicy	2.20E-02	Ester
43	Isopentyl hexanoate	2198-61-0	1250	fruity, banana, apple	3.20E-01	Ester
44	1-Butanol, 3-methyl-, formate	110-45-2	792	plum, black currant, ethereal	1.49E+02	Ester
45	Ethyl 9-decenoate	67233-91-4	1388	fruity, fatty	2.00E-01	Ester

CAS: Chemical Abstracts Service Registry Number; NIST: National Institute of Standards and Technology; RI: Retention Index.
