# Supplementary material for: Dynamic Change of Volatile Fatty Acid Derivatives (VFADs) and Their Related Genes Analysis during Innovative Black Tea Processing
Source: Foods. 2024 Sep 28;13(19):3108. doi: 10.3390/foods13193108 (PMC11475071; doi:10.3390/foods13193108)
Supplement: Supplementary file 1 [file foods-13-03108-s001.zip › Table S3.pdf]

Table S3. Total RNA quality information of samples

Samples	Concentration(ng/μl)	OD260/280	OD260/280	Total amount(ng)
L-1	381.70	2.04	1.60	13.36
L-2	529.30	2.15	0.93	18.53
L-3	774.80	2.16	1.89	27.12
W-1	959.30	1.85	0.44	33.58
W-2	1050.70	2.08	1.15	36.77
W-3	615.90	2.12	1.66	21.56
T-1	808.50	2.11	1.98	28.30
T-2	824.20	2.05	1.70	28.85
T-3	794.70	2.10	1.78	27.81
S-1	1383.60	2.06	1.85	48.43
S-2	834.50	2.09	1.53	28.21
S-3	1254.40	2.08	1.98	43.90
F-1	611.00	2.16	2.18	21.39
F-2	98.70	2.03	2.12	17.45
F-3	601.20	2.18	2.25	21.04
